# Treadmill Exercise Attenuates Retinal Oxidative Stress in Naturally-Aged Mice: An Immunohistochemical Study

**DOI:** 10.3390/ijms160921008

**Published:** 2015-09-02

**Authors:** Chan-Sik Kim, Sok Park, Yoonseok Chun, Wook Song, Hee-Jae Kim, Junghyun Kim

**Affiliations:** 1 Korean Medicine Convergence Research Division, Korea Institute of Oriental Medicine, Daejeon 34054, Korea; E-Mail: chskim@kiom.re.kr; 2 Department of Sports and Health Management, Mokwon University, Daejeon 35349, Korea; E-Mail: sok@ajou.ac.kr; 3 Sports Wellness Center, Yong In University, Gyeonggi-do 17092, Korea; E-Mail: chjordan@naver.com; 4 Health and Exercise Science Laboratory, Seoul National University, Seoul 08826, Korea; E-Mails: songw3@snu.ac.kr (W.S.); skim82@snu.ac.kr (H.-J.K.)

**Keywords:** aging, exercise, oxidative stress, retina, treadmill

## Abstract

In the retina, a number of degenerative diseases, including glaucoma, diabetic retinopathy, and age-related macular degeneration, may occur as a result of aging. Oxidative damage is believed to contribute to the pathogenesis of aging as well as to age-related retinal disease. Although physiological exercise has been shown to reduce oxidative stress in rats and mice, it is not known whether it has a similar effect in retinal tissues. The aim of this study was to evaluate retinal oxidative stress in naturally-aged mice. In addition, we evaluated the effects of aerobic training on retinal oxidative stress by immunohistochemically evaluating oxidative stress markers. A group of twelve-week-old male mice were not exercised (young control). Two groups of twenty-two-month-old male mice were created: an old control group and a treadmill exercise group. The old control group mice were not exercised. The treadmill exercise group mice ran on a treadmill (5 to 12 m/min, 30 to 60 min/day, 3 days/week for 12 weeks). The retinal thickness and number of cells in the ganglion cell layer of the naturally-aged mice were reduced compared to those in the young control mice. However, treadmill exercise reversed these morphological changes in the retinas. We evaluated retinal expression of carboxymethyllysine (CML), 8-hydroxy-2′-deoxyguanosine (8-OHdG) and nitrotyrosine. The retinas from the aged mice showed increased CML, 8-OHdG, and nitrotyrosine immunostaining intensities compared to young control mice. The exercise group exhibited significantly lower CML levels and nitro-oxidative stress than the old control group. These results suggest that regular exercise can reduce retinal oxidative stress and that physiological exercise may be distinctly advantageous in reducing retinal oxidative stress.

## 1. Introduction

Aging is a complex physiological phenomenon that is defined as a progressive loss of the efficacy of physiological and biochemical processes, which occurs until death [1]. Age-related retinal structural and physiologic changes are well documented in elderly persons and animals [2,3]. In the retina, a number of degenerative diseases, including glaucoma, diabetic retinopathy, and age-related macular degeneration, may occur in the course of aging. The retina is highly vulnerable to oxidative injury induced by reactive oxygen species [4]. Retinal photoreceptor cells are among the most metabolically active cells, and they do not divide. Thus, these cells are particularly sensitive to oxidative damage due to their increased oxygen consumption [5]. In retinal pigment epithelial cells, exposure to high doses of short wavelength visible light is cumulative, resulting in aging-associated retinal dysfunction, visual decline, or even vision loss, such as that associated with age-related macular degeneration, which is the leading cause of blindness among the elderly [6]. Although the specific mechanisms involved in the initiation of age-related retinal disease remain unknown, it is believed that oxidative stress is an important causative factor in the development and progression of these diseases [7]. Retinal cells are exposed to a cumulative amount of oxidative and metabolic stress during the aging process. Increased oxidative stress and the accumulation of oxidative-modifications in key molecules induce the dysfunction of signaling pathways in various cells, thereby causing retinal cell injury [8]. There is still a lack of an efficacious remedy for these age-related retinal diseases due to incomplete understanding of the underlying mechanisms.

Physical exercise is known to reduce oxidative stress [9,10]. Moderate exercise reduced oxidative stress by inducing the expression of antioxidant enzymes [11]. Treadmill exercise prevented diabetes-induced apoptosis in retinal cells in rats [12] and protected retinal cells against light-induced retinal cell degeneration [13]. However, few studies have assessed retinal oxidative stress [8,14] and the beneficial effects of exercise in aging retinas [15].

Thus, in the present study, we evaluated retinal oxidative stress in naturally-aged mice. In addition, we examined the effects of aerobic training on retinal oxidative stress by immunohistochemically evaluating oxidative stress markers.

## 2. Results

### 2.1. Histopathology

**Figure 1:**
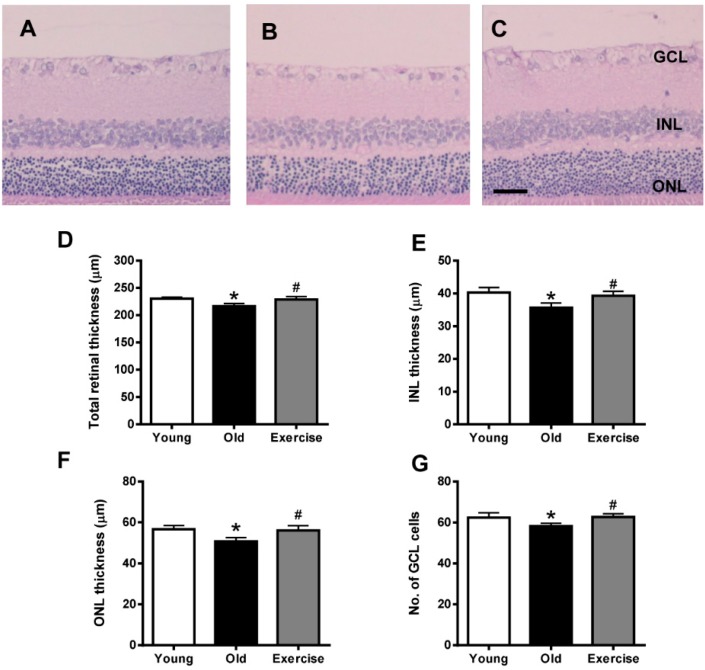
Histopathological retinal changes. Representative retinas from the young control (**A**); old control (**B**); and exercise (**C**) groups were stained with H&E. Retinal thickness of the total retina (**D**); the inner nuclear layer (INL, **E**); and the outer nuclear layer (ONL, **F**); (**G**) Quantification of the cell loss in the ganglion cell layer (GCL), as assessed by the number of cells in the GCL. The values in the bar graphs represent the means ± SE, *n* = 8. * *p* < 0.05 *vs*. the young control group; # *p* < 0.05 *vs*. the old control group. Scale bar = 50 μm.

In histopathological examination of hematoxylin and eosin (H&E)-stained retinal sections (Figure 1), we found that the retinal thicknesses of naturally aged mice (216.7 ± 14.8 µm) were slightly less than those in the young control mice (230.0 ± 10.0 µm, *p* < 0.05). Specifically, the thicknesses of the outer nuclear layer (ONL) and inner nuclear layers (INL) were significantly reduced in the aged mice (ONL: 50.6 ± 6.0 µm, INL: 35.6 ± 4.4 µm) compared to those in the young control mice (ONL: 56.6 ± 5.7 µm, INL: 40.2 ± 5.0 µm, *p* < 0.05). We also measured the number of cells in the ganglion cell layer (GCL). In the naturally-aged mice, there were slightly fewer cells (58.2 ± 4.4) in the GCL compared to the young control mice (62.4 ± 7.1, *p* < 0.05). However, treadmill exercise reversed these morphological changes (total retinas: 229.0 ± 16.3 µm, ONL: 56.1 ± 7.3 µm, INL: 39.3 ± 4.3 µm, and the number of cells in the GCL: 62.7 ± 4.9, *p* <0.05) in the retinas.

### 2.2. Changes in Oxidative Stress Markers in the Renal Tissues

To determine whether treadmill exercise reduced retinal oxidative stress, we examined the immunohistochemical staining of carboxymethyllysine (CML), 8-hydroxy-2′-deoxyguanosine (8-OHdG) and nitrotyrosine. CML is a general marker of local oxidative stress and tissue damage due to protein oxidation [16]. The oxidation of guanine to form 8-OHdG is a marker of oxidative DNA damage [17]. The formation of nitrotyrosine is regarded as a marker of nitro-oxidative stress and is observed when excess nitric oxide and oxidants are produced [18].

**Figure 2:**
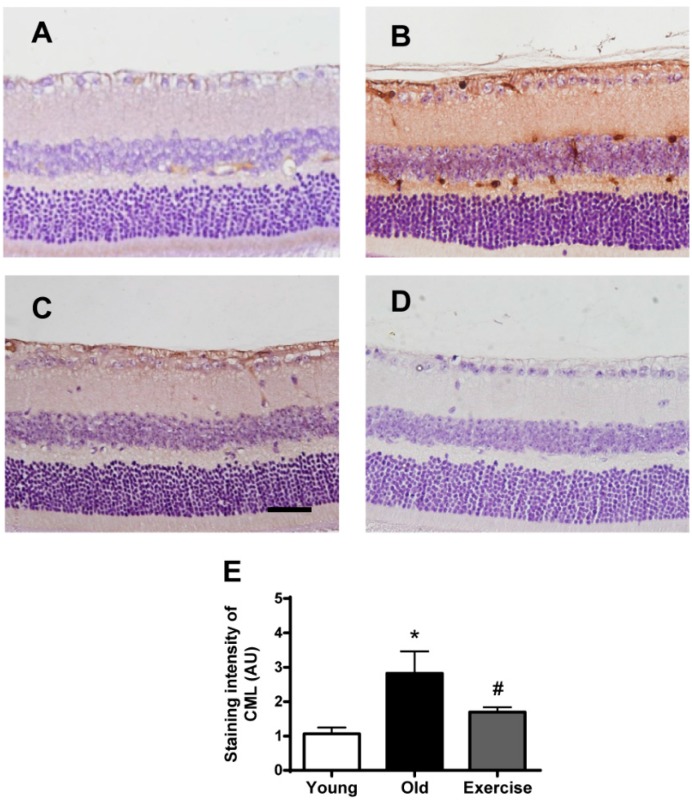
Retinal carboxymethyllysine (CML) formation. Immunohistochemical staining for CML, a local oxidative stress marker, was performed on the retinas from the young control (**A**); old control (**B**); exercise (**C**) groups; and (**D**) negative control. The primary antibody was omitted. Scale bar = 50 μm. CML immunoreactivity was only observed in the large and small retinal vessels of the young control mice, whereas CML-positive signals were found in both the retinal vessels and the inner neural retina in the old control mice. However, exercise reduced CML formation in these regions; and (**E**) the values in the bar graphs represent the means ± SE, *n* = 8. The staining intensity results are reported in arbitrary units (AU). * *p* < 0.05 *vs*. the young control group; # *p* < 0.05 *vs*. the old control group.

As shown in Figure 2, retinal CML immunoreactivity was significantly higher in the aged mice compared to the young control mice. CML expression was primarily distributed in the retinal vessels and the inner neural retina, indicating that oxidative stress induces CML formation in the retina during the aging process. Treadmill training prevented CML formation in these regions of the retina. In quantitative analysis, the level of CML immunoreactivity was increased three-fold (2.8 ± 1.4) in the aged mice compared to the young control mice (1.0 ± 0.4). These changes were significantly reduced by treadmill exercise (1.7 ± 0.3, *p* < 0.05).

**Figure 3:**
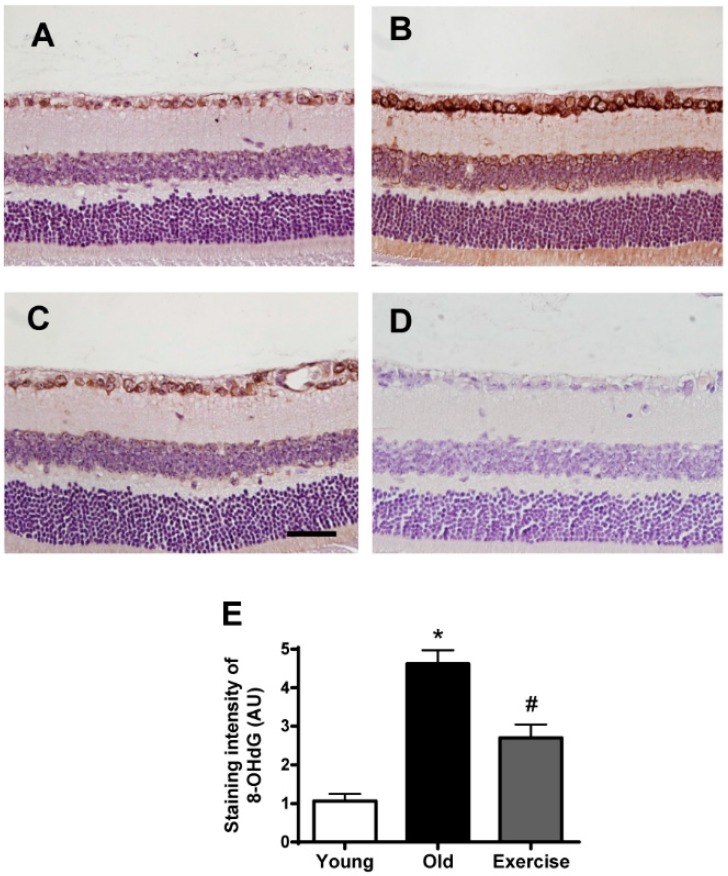
Retinal 8-hydroxy-2′-deoxyguanosine (8-OHdG) formation. Immunohistochemical staining for 8-OHdG, an oxidative DNA damage marker, was performed on the retinas from the young control (**A**); old control (**B**); exercise (**C**) groups; and (**D**) negative control. The primary antibody was omitted. Scale bar = 50 μm. 8-OHdG immunoreactivity was weakly detected in the ganglion cell layer of the young control mice, whereas 8-OHdG-positive signals were observed in the nuclei of the ganglion cell layer and the inner nuclear layer in the old control mice. However, exercise reduced 8-OHdG formation in retinal tissues; and (**E**) the values in the bar graphs represent the means ± SE, *n* = 8. * *p* < 0.05 *vs*. the young control group; # *p* < 0.05 *vs.* the old control group.

Similarly, retinal sections from the aged and young control mice were immunohistochemically stained for 8-OHdG (Figure 3). In the young control mice, weak immunoreactivity for 8-OHdG was detected in a few cells of the ganglion cell layer, but no immunoreactivity was detected in the inner and outer nuclear layers. However, enhanced 8-OHdG immunoreactivity was detected in the nuclei within the ganglion cell layer, the inner nuclear layer, and the outer nuclear layer of the aged mice, indicating increased oxidative DNA damage. Treadmill training reduced the retinal oxidative DNA damage in these regions. In quantitative analysis, the immunostaining intensity of 8-OHdG was increased four-fold (4.6 ± 0.7) in the aged mice compared to the young control mice (1.0 ± 0.3) and was significantly decreased in exercised mice (2.7 ± 0.7, *p* < 0.05).

**Figure 4:**
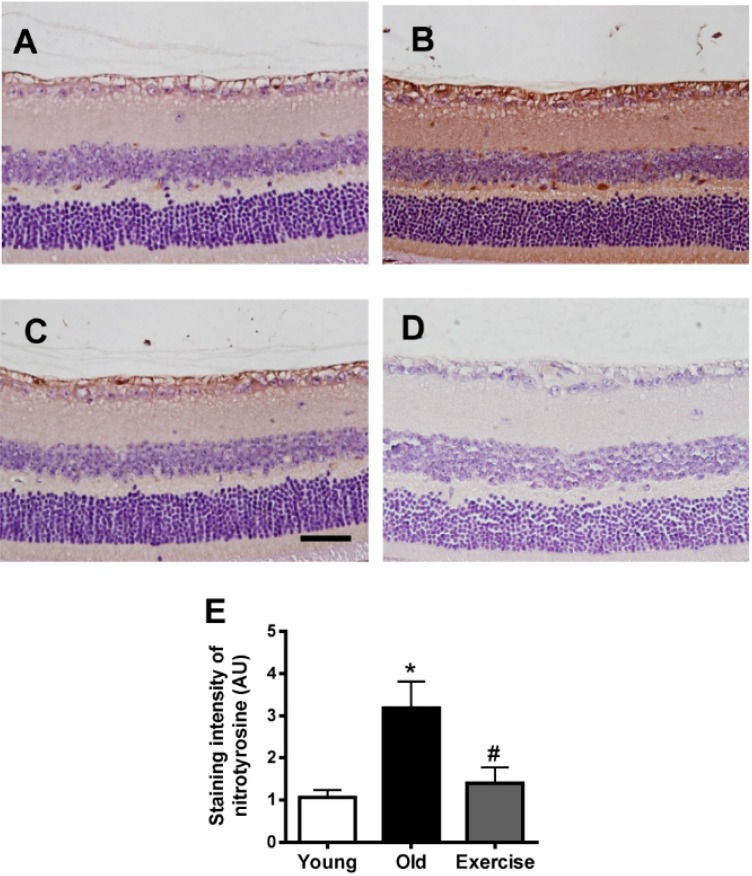
Retinal nitrotyrosine formation. Immunohistochemical staining for nitrotyrosine, a nitro-oxidative stress maker, was performed on the retinas from the young control (**A**); old control (**B**); exercise (**C**) groups; and (**D**) negative control. The primary antibody was omitted. Scale bar = 50 μm. Nitrotyrosine was barely detectable in the young control mice, but the old control mice displayed enhanced nitrotyrosine immunoreactivity in retinal tissues. Exercise significantly inhibited nitrotyrosine formation; and (**E**) the values in the bar graphs represent the means ± SE, *n* = 8. * *p* < 0.05 *vs*. the young control group; # *p* < 0.05 *vs*. the old control group.

Immunohistochemical staining for nitrotyrosine is shown in Figure 4. Weak immunoreactivity for nitrotyrosine was observed in the young control retinas, but a significant increase in nitrotyrosine staining intensity was widely detected in all retinal cell layers of the old control mice. Treadmill exercise suppressed nitrotyrosine expression compared to the old control group. In quantitative analysis, the exercised mice had significantly reduced retinal nitrotyrosine formation (1.4 ± 0.8) compared to the old control group (3.1 ± 1.4, *p* < 0.05).

These findings suggest that oxidative retinal injury occurred in naturally-aged mice and that treadmill exercise may have inhibited age-related oxidative retinal damage.

## 3. Discussion

Our immunohistochemical study provided evidence indicating that enhanced neuronal damage occurred in the retinas of naturally-aged mice. Histopathological study also showed that structural alterations were significantly induced in the retinal cells of aged mice compared to young control mice. These findings indicate that significant oxidative stress in the retinas led to significant neuronal loss. This reduction in the number of retinal neurons is in agreement with the results of Samuel *et al*. [3], who observed a small decrease in the number of retinal ganglion cells and photoreceptor cells in 26-month-old mice compared to young controls.

In addition, we showed that treadmill exercise exerts retinoprotective effects in naturally-aged mice. Similarly, Chrysostomou *et al.* reported that swimming exercise ameliorated acute pressure-induced optic nerve injury in aged mice (12-month-old) [15]. To the best of our knowledge, our data describe the first link between age-related neuronal cell loss and oxidative stress in retinal tissues and provide the first direct evidence that exercise could reduce retinal oxidative stress in the aging process.

Consistent with our results, an age-dependent increase in CML formation in retinal tissues has been observed in human subjects [19]. Twelve-month-old SOD1^−/−^ mice, which are used as an animal model of age-related macular degeneration, displayed increased CML formation in Bruch’s membrane and in the pigment epithelium compared to age-matched, wild-type mice [20]. CML is a major product of the oxidative modification of glycated proteins. The glycoxidation reaction to generate CML only occurs in the presence of superoxide radicals and hydrogen peroxide [21]. Moreover, the formation of CML is irreversible. Thus, CML may be an integrative biomarker of accumulated oxidative stress [22]. CML is known to produce carbonyl and oxidative stress, which may cause both direct and receptor-mediated injury [23]. Our results showed that aged retinas displayed a significant increase in CML formation. This finding indicates that the reduced retinal thickness observed in aged retinas might have been partially mediated by CML-induced cellular injury.

Similarly, extensive retinal 8-OHdG formation has been demonstrated in aged mice (22–26 months) [14], which is consistent with our results. Vitreal 8-OHdG levels were significantly increased in patients with age-related macular degeneration [24]. 8-OHdG is one of the most abundant products of oxidative DNA damage and represents a sensitive and stable biomarker of oxidative stress. Age-related increases in 8-OHdG formation have been found in various human organs [25,26]. Our results also showed an age-related increase in oxidative DNA damage in aged retinas, providing evidence that oxidative DNA damage is associated with retinal aging. Liles *et al.* reported that human eye specimens had decreased antioxidant enzyme activity with aging [27]. Unfortunately, we could not measure the reactive oxygen species (ROS) content in the eye. However, it has been reported that a linear relationship exists between ROS production and 8-OHdG formation [28]. Thus, it is reasonable to assume that the increased formation of 8-OHdG in aged mice might be attributed to its influence on ROS production.

Physical exercise has a positive influence on oxidative status [29,30] and improves oxidative stress parameters by reducing oxidant production [31]. In addition, exercise-induced increased energy demands might decrease the pool of reactive intermediates for glycoxidation or lipoxidation [32]. Here, we hypothesized that the reduction in oxidative stress following treadmill exercise may contribute to the inhibition of structural alterations in the retina. The current study clearly demonstrated that treadmill exercise restored the expression of oxidative stress markers to near-normal levels in the naturally-aged mice, which paralleled the marked inhibition of neuronal cell loss. Recently, Tsou *et al.* showed that treadmill exercise protected dopaminergic neurons by regulating the nuclear factor erythroid 2-related factor 2 (Nrf2) antioxidant system in a 1-methyl-4-phenylpyridine-induced Parkinsonian rat model [33]. Similarly, exercise increased superoxide dismutase (SOD, an anti-oxidant defense marker) activity and nuclear levels of Nrf2 in renal proximal tubules of old rats (23 months old) [9]. These findings provide evidence that physical exercise confers antioxidant effects in the retina; these antioxidant effects may account for the beneficial effect of exercise on age-related retinal degeneration.

Reactive oxygen species or reactive nitrogen species are produced during normal physiological processes, but their production is accelerated under disease conditions and during the aging process [8]. Reactive nitrogen species such as nitric oxide play an important role in the development of glaucoma, ischemia-induced retinopathy, and diabetic retinopathy [34,35,36]. In the retina, reactive nitrogen species can trigger nitro-oxidative damage of proteins, lipids, and DNA, resulting in photoreceptor cell death, which is involved in the pathogenesis of age-related macular degeneration [37]. Xu *et al.* showed that retinal nitrotyrosine formation was markedly increased in 24-month-old mice [8], similar to our present results. These findings suggest that age-related nitro-oxidative stress is also involved in the retinal aging process. Exercise reduced plasma nitrotyrosine levels in patients with rheumatoid arthritis [38]. Additionally, regular physical exercise decreased nitrotyrosine production in the skeletal muscle of patients with chronic heart failure [39]. Consistent with a previous reports, we also showed that nitrotyrosine formation was increased in aged retinas. The reduction of nitrotyrosine formation following treadmill exercise was associated with a suppression of age-related retinal structural changes.

In this study, we did not include an exercise group of young mice. The reason for the omission is that young mice had no histological and functional changes in the retina. Wang *et al.* showed that oxidative mitochondrial DNA damage is initiated in retinal pigment epithelial cells and choroid at 18 months of age [14]. In another previous study, aged mice (24–28 months old) exhibited thin retinas, and the number of retinal ganglion cells was decreased compared to young mice (3–5 months old). However, the electrophysiological function is preserved in old retinal ganglion cells [3]. Moreover, treadmill exercise in normal adult mice induced no significant difference in the number of retinal photoreceptor cells compared to adult mice without exercise [13]. Based on these results, we omitted a young exercise group.

In summary, our study demonstrates that treadmill exercise has anti-oxidative effects in the retinas of naturally-aged mice. These novel findings provide insights into the retinoprotective effects of regular physical exercise.

## 4. Experimental Section

### 4.1. Animals and Experimental Design

Male C57BL/6J mice were purchased from Orient Bio (Seoul, Korea). Twelve-week-old mice were not exercised (young control). Twenty-two-month-old male mice were divided into old control and aerobic exercise groups. The old control mice were not exercised. The mice in the exercise group were forced to run on a motor-driven treadmill once a day, three times per week for 12 weeks. The exercise training protocol was performed according to a previously-described method with some modifications [40]. On the first day, the animals were placed in the treadmill chamber and allowed to acclimate for 20 min. Each day, the animals were given a 3–5 min warm-up period with slow walking speeds. Training began at a pace of 5 m/min for 30 min. As the mice became increasingly familiar with the treadmill, their velocities were gradually increased until they were able to run at 12 m/min for 60 min. Generally, both the pace and time were increased in an attempt to achieve a ~10% increase per week. A pace of 12 m/min is considered to be a moderate walk-jog pace for mice [41]. The mice were constantly monitored while they were on the treadmill. If a mouse appeared to be injured during the training, it was immediately removed from the treadmill. At necropsy, both eyes from each mouse were enucleated under deep anesthesia following an intraperitoneal injection of pentobarbital sodium (30 mg/kg body weight). For conventional histopathological examinations, the left eyes were fixed in 10% neutralized formalin for 24 h and then embedded in paraffin. The right eyes were snap-frozen in OCT compound (Tissue-Tek, Sakura Finetek, Tokyo, Japan) and cryosectioned at a 6-μm thickness. All experiments were approved by our Institutional Animal Care and Use Committee (Daejeon, Korea).

### 4.2. Histopathology

The formalin-fixed whole eyes were embedded in paraffin and sagittally sectioned (4 μm); the sections were then stained with H&E. The thickness of the retinal layers was measured in a region 0.5 mm from the optic disc using image analysis software (ImageJ, NIH, Maryland, MD, USA). The total number of cells in the GCL was counted in an approximately 1 mm linear region of GCL from the optic nerve head. The counts from the two sides were averaged.

### 4.3. Immunohistochemical Staining for Oxidative Stress Markers

The eye sections were prepared on a cryostat and mounted onto glass slides coated with poly-l-lysine. The eye sections were permeabilized by incubating them in 0.2% Triton X-100 in PBS containing 1% bovine serum albumin. Immunohistochemistry was performed as previously described [42]. The following antibodies were used: mouse anti-CML (1:250, TransGenic, Kobe, Japan), mouse anti-8-OHdG (1:100, Santa Cruz Biotechnology, California, CA, USA), and rabbit anti-nitrotyrosine (1:500, Santa Cruz Biotechnology, California, CA, USA). For the detection of CML, 8-OHdG, and nitrotyrosine, the sections were incubated with Envision kit reagents (Dako, California, CA, USA) and visualized with 3,3′-diaminobenzidine tetrahydrochloride. The negative controls for immunohistochemistry were performed by incubating the sections with nonimmune serum rather than with primary antibody. The images were captured using an Olympus BX51 microscope with a DP71 digital camera (Olympus, Tokyo, Japan). After the immunohistochemical procedures, the slides were randomly coded and examined in a blind approach. The intensity of the immunohistochemical staining was analyzed by comparing each retinal sections from the central and midperipheral retinal areas of eight animals per group using an image analysis system (ImageJ, NIH, Maryland, MD, USA). Positively-labeled cells were identified using the standard color threshold algorithm of ImageJ software (NIH, Maryland, MD, USA). The pixel intensity and area were then measured, and the data were expressed as the immunoreactivity intensity per field of the selected region.

### 4.4. Statistical Analysis

The data are expressed as the means ± standard error of the mean (SE). Statistical significance was analyzed using Prism 6.0 software (GraphPad, La Jolla, CA, USA). Semi-quantitative data were considered to be non-parametric. Therefore, they were analyzed using the non-parametric Kruskal-Wallis test. When the retinal thickness and cell number were compared, statistical significance was analyzed using one-way ANOVA and Tukey’s multiple comparison test. *p* < 0.05 was considered statistically significant.
